# Emerging Circulating Biomarkers in Atherosclerosis: From Molecular Mechanisms to Therapeutic Strategies

**DOI:** 10.3390/biom12060809

**Published:** 2022-06-09

**Authors:** Giuseppe Mandraffino, Alessandro Mattina, Roberto Scicali

**Affiliations:** 1 Internal Medicine Unit, Lipid Center, Department of Clinical and Experimental Medicine, University of Messina, 98125 Messina, Italy; 2 Diabetes and Islet Transplantation Unit, Department of Diagnostic and Therapeutic Services, IRCCS-ISMETT (Istituto Mediterraneo per i Trapianti e Terapie ad Alta Specializzazione), UPMC Italy, 90127 Palermo, Italy; alessandromattina@gmail.com; 3 Department of Clinical and Experimental Medicine, University of Catania, 95124 Catania, Italy; robertoscicali@gmail.com

Atherosclerosis is a long-term damaging process, and its progression leads to cardiovascular system injury. Several environmental and genetic factors play a critical role in atherosclerosis progression; among these, an increased amount of low-density lipoprotein (LDL) cholesterol (LDL-C) plasma level is causatively associated with atherosclerotic cardiovascular disease (ASCVD). However, the full definition of atherosclerosis, as the chronic storage of LDL-C, may not be exhaustive; in fact, further processes are involved in the pathogenesis and progression of atherosclerotic injury together with LDL cholesterol. In the last few years, several studies have indicated the importance of inflammatory status in the pathophysiology of ASCVD; in particular, inflammation appears to be the final expression of the systemic interaction between the amount of cholesterol and the immune system during atherosclerotic progression. In subjects with dyslipidemia, previous studies have shown that lipid storage in the arterial wall promotes the inflammatory cascade and thus the migration of immune cells, such as monocyte-derived macrophages and T lymphocyte subtypes, into inflammatory lipid wall injuries. The concept of atherosclerosis as a continuous inflammatory process promoted by a persistent amount of LDL-C and immune system activation may explain why, despite lifestyle changes and lipid-lowering treatments, ASCVD is still considered the leading cause of death and disease burden. In this context, novel circulating biomarkers may be helpful to better define the link between lipid metabolism, inflammation, and the immune system in the atherosclerotic process in the general population, as well as—in particular—in subjects with a high risk of cardiovascular events [1,2,3].

The composition and structure of lipoproteins are associated with their atherogenic potential; Rehues et al. focused on the content of LPS, and of its immunogenic portion, 3OHFA, in lipoprotein fractions and particles. They showed significant levels of LPS in HDL, LDL, and also in VLDL, suggesting a potential contribution of LPS and 3OHFA to atherogenesis in humans. Upcoming studies evaluating the utility of LPS in lipoproteins as a biomarker of atherosclerosis are awaited [4].

In their experimental model, Manjarrez-Reyna et al. aimed to investigate the effect of native LDL on the immune function of human monocyte subpopulations in in vitro LPS-stimulated primary monocytes and in patients with high LDL-C levels as well as LPS-binding protein serum levels; they suggest a model where the native LDL acts in synergy with LPS, inducing the mononuclear cells to produce inflammatory cytokines and chemokine receptors with prominent roles in atherogenesis [5].

Ugovšek and Šebeštjen aimed to refine, with their literature review, some interesting pathophysiological aspects about the role of lipoprotein(a) as a proatherosclerotic, proinflammatory, prothrombotic, and antifibrinolytic player: what does it do at the “Crossroads of atherosclerosis, atherothrombosis and inflammation”? Additionally, what can we modify through lowering Lp(a) [6]?

Chronic, often low-graded inflammation is a common feature of inflammatory rheumatic diseases (IRDs); IRDs are burdened by a pronounced incidence of ASCVD that is 1.5-fold greater than that seen in non-IRD patients, and novel as well as effective tools are needed to stratify CV risk and evaluate the impacts of therapies [7,8,9]. Czókolyová et al. evaluated the effects of TNF inhibition on circulating metabolic biomarkers—including paraoxonase activity, arylesterase activity, myeloperoxidase, leptin, adiponectin, and chemerin levels—in rheumatoid arthritis and ankylosing spondylitis patients, in addition to the associations between these detectable indices with disease activity and vascular parameters. They concluded that one year of anti-TNF treatment significantly improved several study parameters associated with vascular function, although lipid fractions did not change between baseline and 12 months [10].

Cardiorenal syndrome (CRS) is a multiorgan disease characterized by the complex interaction between the heart and kidney during acute or chronic injury; Gembillo et al. aimed to investigate, through a comprehensive review, which biomarkers could be of help in the diagnosis, risk assessment, and prognosis of CRS [11].

Growing evidence is confirming angiopoietin-like 3 (ANGPTL3) as a key player in lipid and lipoprotein metabolism, able to inhibit both lipoproteins and endothelial lipases, thus playing a relevant role in the regulation of FFA turnover and partitioning. Through a charming experimental design, Bini et al. aimed to increase the current knowledge on the direct effect of ANGPTL3 on adipocytes, opening a new way to consider it as a very promising therapy target [12].

PCSK9, proprotein convertase subtilisin/kexin type 9, is an unquestionable target of the most effective therapy to reduce plasma lipids and CV risk; indeed, could PCSK9 also represent something further? Toscano et al. investigated the potential role of PCSK9 in the primary prevention of heterozygous familial hypercholesterolemia (HeFH) subjects at baseline and after therapy optimization by high-efficacy statin, ezetimibe, and PCSK9-inhibiting monoclonal antibodies. They found that PCSK9 plasma levels are correlated with PWV at baseline, and that the decrease in PCSK9 levels is associated with a mechanical vascular improvement after PCSK9-i therapy. These results could suggest PCSK9 as a potential biomarker of vascular function in high-CV-risk patients, as well as a potential marker of CV residual risk when the LDL-C goal is attained [13].

In the era of personalized medicine, precision tools are required to significantly improve the quality of clinical assistance, and integrative approaches are needed to develop what we lack to date. Usova et al. proposed a new and very innovative approach to atherosclerotic cardiovascular risk prediction through the “integrative analysis of multi-omics and genetic approaches”; by blending multiomics, genetic approaches, and clinical features, in fact, the aim of their research is to provide clinicians with effective predictive tools of personalized medicine to be applied at any stage of ACVD risk [14].

This Special Issue is focused on the role of novel circulating biomarkers in atherosclerosis through the description of molecular mechanisms and the modulation of innovative therapies. CVD risk assessment, prediction, and modification are continuously evolving topics; the biomarkers, players, pathways, and therapies of today should be the cornerstone of the knowledge of tomorrow.

## Data Availability

Not applicable.
